# Predicting adolescent disordered eating and behaviours: exploring environmental moderators of polygenic risk

**DOI:** 10.1111/jcpp.70012

**Published:** 2025-07-09

**Authors:** Madeleine Curtis, Lucia Colodro‐Conde, Sarah E. Medland, Scott Gordon, Nicholas G. Martin, Tracey D. Wade, Sarah Cohen‐Woods

**Affiliations:** ^1^ Discipline of Psychology, College of Education, Psychology, and Social Work Flinders University Adelaide SA Australia; ^2^ Blackbird Initiative, Flinders Institute for Mental Health and Wellbeing Flinders University Adelaide SA Australia; ^3^ QIMR Berghofer Medical Research Institute Brisbane Qld Australia; ^4^ School of Psychology The University of Queensland Brisbane Qld Australia; ^5^ Flinders Centre for Innovation in Cancer, College of Medicine and Public Health Flinders University Adelaide SA Australia

**Keywords:** Eating disorder, behavioural genetics, gene‐environment interaction (GxE), anorexia nervosa

## Abstract

**Background:**

Both genetic and environmental factors contribute to the risk of developing disordered eating, with twin studies demonstrating environmental factors moderate genetic susceptibility. To date, gene–environment interactions leveraging polygenic risk scores (PRS) have not been studied in disordered eating phenotypes beyond anorexia nervosa (AN). This study investigated if polygenic risk for AN interacts with established environmental eating disorder risk factors (parental expectations, parental criticism, parental conflict, parental care and weight‐related peer teasing) to predict overall levels of disordered eating in the general population or specific lifetime disordered eating behaviours (avoidance of eating, objective bulimic episodes, self‐induced vomiting and driven exercise).

**Methods:**

PRS were calculated using summary statistics from the largest AN genome‐wide association study. Environmental factors were assessed via telephone interview using standardized measures. Analyses were performed using genome‐wide complex trait analysis to test whether parental expectations, criticism, conflict or care, or weight‐related peer teasing interacted with AN PRS to predict disordered eating outcomes in our sample (*n* = 383).

**Results:**

The analyses revealed significant main effects of parental expectations, parental criticism, parental care, and weight‐related peer teasing on at least one disordered eating outcome. All environmental variables moderated the association between AN PRS and at least one disordered eating outcome by either increasing risk (parental expectations, parental criticism, parental conflict, weight‐related peer teasing) or lowering risk (parental care).

**Conclusions:**

Findings highlight the complex interplay between genetic and environmental factors in disordered eating development and emphasize the importance of personalized interventions that consider both genetic predisposition and environmental influences.

## Introduction

Disordered eating encompasses both clinical eating disorders, as well as disordered eating‐related behaviours and cognitions (e.g. intense fear of weight gain, food restriction, self‐induced vomiting) that may not meet DSM‐5 diagnostic criteria, but still cause significant distress and impairment (American Psychiatric Association [APA], 2022). Genetic factors play a significant role in the development of disordered eating, with heritability estimates from twin studies ranging from 16% to 83% for clinical eating disorders (Wade & Bulik, 2018; Watson et al., 2021), and 32%–72% for specific disordered eating symptoms (Berrettini, 2004; Yilmaz, Hardaway, & Bulik, 2015). Twin studies estimate total genetic influence, including gene–environment interactions, which may inflate heritability estimates (Culbert, Racine, & Klump, 2015), whereas recent genome‐wide association studies (GWAS) provide a more conservative estimate, capturing only the heritability explained by common genetic variants. Current GWASs on anorexia nervosa (AN) estimate that common genetic variation accounts for 11%–17% of the heritability of AN; a figure that is expected to increase with larger samples and improved statistical power (Hübel, Leppä, Breen, & Bulik, 2018; Watson et al., 2019).

Although genetic factors can increase the risk of developing disordered eating, factors such as societal influences and family environment also play an important role (Mitchison & Hay, 2014). Many environmental factors have been associated with disordered eating, including weight‐related peer teasing and parental factors such as parental conflict, parental criticism, parental expectations, and parental care. There is robust evidence for an association between disordered eating and weight‐related peer teasing, with multiple studies reporting positive associations between weight teasing and increased disordered eating outcomes (Fairburn et al., 1998; Fairweather‐Schmidt & Wade, 2015, 2017; Menzel et al., 2010; Neumark‐Sztainer et al., 2007; Wade & O'Shea, 2015). A meta‐analysis by Menzel et al. (2010) reported a medium to large effect for the relationship between weight teasing and body dissatisfaction, and a moderate association between weight teasing and both dietary restraint and bulimic behaviours. This relationship was stronger in children and adolescents compared to adults (Menzel et al., 2010). Additionally, research has shown that both experiencing an increase in weight‐related peer teasing from early to mid‐adolescence and higher levels of weight‐related peer teasing during mid‐adolescence were linked to the development of disordered eating in late adolescence (Fairweather‐Schmidt & Wade, 2015). The effect of weight‐related peer teasing on disordered eating can also be long‐lasting, with a longitudinal study reporting that girls who were teased about their weight were at 1.5 times the risk of engaging in binge eating or extreme weight control behaviours 5 years later (Neumark‐Sztainer et al., 2007). Together, these findings underscore adolescence as a critical period for the influence of peer interactions on disordered eating behaviours.

Parental conflict, specifically referring to conflict between parents, has been associated with various disordered eating outcomes (Blodgett Salafia, Schaefer, & Haugen, 2014; Fairburn, Cooper, Doll, & Welch, 1999; Wade, Bulik, & Kendler, 2001; Wade, Gillespie, & Martin, 2007). A study by Wade et al. (2007) reported that in twins discordant for bulimia nervosa (BN), those with BN reported higher parental conflict compared to their healthy twins. Similarly, research demonstrates that individuals with AN report more parental arguments compared to healthy controls (Fairburn et al., 1999). Individuals with an eating disorder (AN, BN, BED) have also reported receiving more general parental criticism compared to healthy controls (Fairburn et al., 1998, 1999; Fairburn, Welch, Doll, Davies, & O'Connor, 1997; Wade et al., 2007), as well as more critical comments from parents about their weight, shape or eating specifically (Machado et al., 2014; Pike et al., 2008). Lower parental care, reflecting less parental warmth and affection, has been associated with disordered eating behaviours as well as diagnoses of AN, BN and BED (Fairburn et al., 1997, 1998, 1999; Micali et al., 2017; Neumark‐Sztainer, Story, Hannan, Beuhring, & Resnick, 2000). Micali et al. (2017) identified that women who reported low maternal care had an increased risk of developing binge/purge type eating disorders, while those reporting high maternal care had a 20% decreased risk of developing BN. This suggests that, in addition to low parental care being a risk factor for disordered eating, high parental care may have a protective effect (Micali et al., 2017). General parental expectations have also been associated with disordered eating; however, the direction of findings has been mixed. Several studies have found high parental expectations to be associated with clinical eating disorders, including AN, BN and BED (Fairburn et al., 1997, 1998, 1999; Karwautz et al., 2001; Wade et al., 2007). In contrast, a study by Neumark‐Sztainer et al. (2000) reported that low parental expectations were associated with an increase in overall disordered eating; however, this discrepancy could be due to the use of a single‐item measure of parental expectations, contrasting with the more comprehensive measures included in the other studies.

Whilst both genetic and environmental factors contribute to disordered eating risk, they have also been proposed to interact to further increase, or protect against, the risk of development of eating pathology. However, few studies have explored gene–environment interactions with parental factors or weight‐related peer teasing, and these have been restricted to twin and candidate gene methods. For example, a twin study by Fairweather‐Schmidt and Wade (2017) demonstrated evidence for a gene–environment interaction with weight‐related peer teasing, whereby genetic influences for dietary restraint, body dissatisfaction, and bulimic behaviours increased when participants experienced weight teasing. The moderating effect of parental factors in individuals with eating disorders was investigated by Karwautz et al. (2011) using a candidate gene approach. The study found that the serotonin transporter *5‐HTTLPR* gene interacted with problematic parenting styles, with genotypes including the short allele (SS or LS) associated with an increased risk of AN in individuals who reported higher parental control, criticism, or expectations.

It is important to note that in addition to gene–environment interactions, gene–environment correlations have been explored in eating disorder research, including both passive correlations where children receive risk genes and environments from parents, and evocative correlations where genetic traits elicit specific environmental responses (Mazzeo & Bulik, 2009). Two recent studies found no evidence of passive gene–environment correlations for disordered eating in girls during pre‐ and early puberty (O'Connor et al., 2022; O'Connor, Burt, McGue, Iacono, & Klump, 2019). However, Wade et al. (2007) reported that among monozygotic twins discordant for BN, the affected twin reported experiencing higher parental expectations compared to their unaffected twin, while Klump, Wonderlich, Lehoux, Lilenfeld, and Bulik (2002) suggested that weight‐related teasing occurring in response to BMI may demonstrate an evocative gene–environment correlation. As the same genetic factors can influence both environmental exposure and outcomes, it is important to account for gene–environment correlations when studying gene–environment interactions.

As eating disorders are polygenic, polygenic risk scores (PRS) have become the preferred measure of genetic risk for complex traits (Hübel et al., 2018). To date, AN is the only eating disorder with sufficiently powered GWAS to generate PRS, though studies for BN and BED are underway (Bulik et al., 2022; Steiger & Booij, 2020). While PRS have been generated for related traits (e.g. BMI), the focus of this study is on understanding shared genetic influences across different disordered eating presentations to reduce multiple testing burden. A recent study by Papini et al. (2024) investigated interactions between AN PRS and several established AN risk factors in predicting AN. Considered individually, Papini et al. (2024) reported a differential effect of AN PRS on AN for (i) males versus females, (ii) paternal income, (iii) individuals born through caesarean section, (iv) maternal income, (v) mothers who experienced a genitourinary tract infection while pregnant (but not other types of infection), (vi) parents education status and (vii) parental history of an eating disorder diagnosis. However, this study did not investigate peer or parental relationship factors.

PRS have not been investigated in the context of gene–environment interactions in eating disorders beyond AN, or disordered eating as a broader phenotype. Given that subclinical disordered eating presentations are associated with substantial impairment (Wilkop, Wade, Keegan, & Cohen‐Woods, 2023), understanding genetic influences on both overall disordered eating and specific behaviours is critical. A previous study by our group demonstrated that AN PRS significantly predicted disordered eating outcomes, including global EDE scores, avoidance of eating, objective bulimic episodes, self‐induced vomiting, and driven exercise, in an Australian adolescent female twin cohort (Curtis et al., 2024). The present study aimed to extend these findings by investigating whether polygenic risk for AN interacts with established environmental risk and protective factors to predict disordered eating behaviours in the same cohort. Specifically, it aimed to test whether weight‐related peer teasing, or parental factors including parental expectations, parental criticism, parental control, and parental care, moderate the relationship between AN PRS and disordered eating global scores, avoidance of eating, objective bulimic episodes, self‐induced vomiting and driven exercise.

## Method

### Sample

Participants included Australian adolescent female–female twin pairs, both MZ and DZ, recruited from the Australian Twin Registry. The data collection process, conducted between 2005 and 2010, has previously been described in detail (Fairweather‐Schmidt & Wade, 2015, 2017, Wade, Byrne, & Bryant‐Waugh, 2008; Wade et al., 2013; Wade & O'Shea, 2015; Wilksch & Wade, 2009). Data were collected longitudinally over three Waves (Wave 1, Wave 2, Wave 3), with participants aged between 12 and 15 years at the first wave of data collection. Wave 1 consisted of 699 participants (mean age 13.96, *SD* = 0.80), Wave 2 included 669 participants (mean age 15.1, *SD* = 0.83), and 499 participants were included in Wave 3 (mean age 16.9, *SD* = 0.70). Participants retained at Wave 3 did not differ significantly from those not retained on key study variables (see Curtis et al., 2024). Waves 1 and 2 consisted of a telephone‐administered interview with the twins and a self‐report questionnaire sent to parents of the twins. Wave 3 consisted of the telephone interview only. Blood samples were collected using EDTA collection tubes from twins at Wave 3 (*n* = 391) for genomic analysis. Response rates were comparable with other large Australian epidemiological twin studies with multiple data collection points (Stice, Marti, & Rohde, 2013; Wade, Bergin, Tiggemann, Bulik, & Fairburn, 2006). Ethical approval was provided by the Flinders University Clinical Research Ethics Committee (no. 115/07) and written informed consent was obtained from all participants.

Participants eligible for inclusion in the present study were those with genomic data available, who responded to the telephone interview measures relevant to each analysis. Eight participants were identified as ancestral outliers and excluded from analyses, leaving a maximum of 383 eligible participants, of whom 229 were monozygotic twins (*n* = 110 complete pairs) and 154 were dizygotic twins (*n* = 75 complete pairs). Participants were of European ancestry and had an average Socioeconomic Indexes for Areas (SEIFA) score of 100.95 (*SD* = 10.80), determined using a standardized measure based on parental income, education, and occupation (*M* = 100, *SD* = 15). The mean age of participants was 14.01 (*SD* = 0.78) at Wave 1, 15.15 (*SD* = 0.82) at Wave 2, and 16.95 (*SD* = 0.83) at Wave 3, with an overall range of 12.74–19.84 years. The present study included 383 participants for analyses involving parental expectations, parental criticism, parental care, and weight‐related peer teasing, and 374 participants for the analysis involving parental conflict. Our analyses were based on molecular genetic methods, and not twin modeling methods. We analyzed AN polygenic risk while controlling for relatedness.

### Measures

The telephone interviews were conducted by postgraduate Clinical Psychology trainees (*n* = 16). Interviews were administered by a different interviewer at separate times for each twin in the family and consisted of two parts. The first part included the 12th edition of the Eating Disorder Examination (EDE) (Fairburn, Cooper, & O'Connor, 1993), administered as a semi‐structured interview and modified slightly for use with children in line with previous recommendations (Bryant‐Waugh, Cooper, Taylor, & Lask, 1996; Wade et al., 2008). Independent ratings of 20 randomly selected interviews demonstrated high interrater reliability across the four EDE subscales, with Pearson's correlations of 1.00 for dietary restraint, .96 for eating concern, .98 for weight concern, and .99 for shape concern (Wade et al., 2008). The second part of the telephone interview included questions from various standardized self‐report questionnaires relating to family functioning, temperament, and attitudes towards weight and appearance.

### Disordered eating

Disordered eating was assessed at each Wave using the EDE (Fairburn et al., 1993). The EDE measures disordered eating over the previous 28 days and consists of 22 items covering four subscales (Dietary Restraint, Eating Concern, Weight Concern, Shape Concern). Items were measured on a 7‐point Likert scale, with scores ranging from 0–6. Global scores were calculated as the mean score across all items. In the present sample, the global EDE scores demonstrated construct and convergent validity, and excellent internal reliability (α = .93 at all Waves) (Fairweather‐Schmidt & Wade, 2015). To ensure the greatest amount of eating pathology experienced by each participant was captured, the present study used the highest global EDE score recorded for each participant across the three Waves of data collection.

The presence of lifetime disordered eating behaviours was also assessed by the EDE using the behavioural frequency items for avoidance of eating, objective bulimic episodes, self‐induced vomiting, and driven exercise. Lifetime behaviours were considered to be present (yes/no) if the participant indicated that there was ever a time when they had engaged with the disordered eating behaviour for a three‐month period. Criteria for each lifetime behaviour are outlined in Table 1. The behavioural frequency questions have shown high interrater reliability across studies, and scores for objective bulimic episodes and self‐induced vomiting have demonstrated test–retest reliability ranging from 0.68 to 0.92 (Berg, Peterson, Frazier, & Crow, 2012).

**Table 1:** Lifetime behaviours assessed in the telephone interview

Behaviour	Criteria to meet threshold
Avoidance of eating	Person has gone for periods of 8 or more waking hours without eating anything in order to influence shape or weight on more than half the days each week for a 3‐month period.
Objective bulimic episodes	Behaviour occurred at least twice a week for a 3‐month period, with breaks of no more than 2 weeks.
Self‐induced vomiting	Behaviour occurred at least twice a week for a 3‐month period, with breaks of no more than 2 weeks.
Driven exercise	Driven or compulsive exercise occurred for at least 1 h, 5 days a week for a 3‐month period, with breaks of no more than 2 weeks.^a^

^a^ Only exercise for weight or shape reasons was included (questions were asked separately for competitive sport and other forms of exercise).

### Parental expectations and parental criticism

Parental expectations and parental criticism were measured at Wave 1 using the Frost Multidimensional Perfectionism Scale (MPS) (Frost, Marten, Lahart, & Rosenblate, 1990). General parental expectations were measured using 5 items derived from the Parental Expectations subscale, and general parental criticism was measured using 4 items taken from the Parental Criticism subscale. Items were measured on a 4‐point Likert scale, where a score of 1 indicated ‘strongly agree’ and a score of 4 indicated ‘strongly disagree’. Total scores for parental expectations and parental criticism were calculated as the mean item score across the respective subscales, with higher reversed totals indicating higher expectations and criticism (range 1–4). Cronbach's alpha was acceptable for both parental expectations (α = .72) and parental criticism (α = .78) (Wilksch & Wade, 2010).

### Parental conflict

Parental conflict was measured at Wave 1 using nine items from the Conflict subscale of the Family Environment Scale (Moos & Moos, 1986). Responses were measured using a 4‐point Likert scale (1 = strongly agree, 4 = strongly disagree). The mean score across items was calculated and used to determine parental conflict scores (range 1–4), with a higher score indicating more conflict. Items were revised to refer to parents rather than families, for example, “My parents fight a lot with each other”. This revised version of the scale has been shown to have good internal reliability in a previous study of adolescent girls (α = .81) (Wade & Lowes, 2002), as well as in the present sample (α = .78) (Wilksch & Wade, 2010).

### Parental care

Parental care was measured at Wave 1 using the Care scale from the Parental Bonding Inventory (PBI) (Parker, Tupling, & Brown, 1979). The scale consisted of 12 items measured on a 4‐point Likert scale where a higher score indicated more care. Scores were calculated as the mean item score for each participant (range 1–4) and were calculated separately for mother's care and father's care. Data on mother's care was reported by 382 participants, and 371 participants reported data on father's care. As mother's care and father's care were strongly correlated (Pearson's *R* = .80), only mother's care is presented in the main text, although analyses for father's care were also undertaken (see Table S2). One participant did not report mother's care, so father's care was used instead. The PBI demonstrated excellent internal consistency in the present sample (α = .91) and has previously shown good test–retest reliability, acceptable construct and convergent validity, and a robust factorial structure across various populations, including adolescents (Parker, 1990; Parker et al., 1979; Wilksch & Wade, 2010).

### Weight‐related peer teasing

Weight‐related peer teasing was measured at Wave 1 using the Weight Teasing – Peers subscale of the McKnight Survey (McKnight Investigators, 2003). The scale consisted of eight items, with four items relating to weight teasing from girls and four items referring to weight teasing from boys. Items were measured on a 5‐point Likert scale (1 = never, 5 = always) where a higher score indicated more weight‐related peer teasing. This subscale showed good internal reliability in the present sample (α = .87).

### Data analysis

DNA samples from the target cohort were genotyped at Erasmus Medical Centre (Rotterdam) using the Infinium Global Screening Array V.1 (Illumina, CA). Raw genotypes were quality‐controlled and imputed using default settings and the HRCr1.1 reference on the Michigan Imputation Server (phased using Eagle; imputed using minimac4). The PRS for AN were calculated using GWAS summary statistics obtained from the Eating Disorders Working Group of the Psychiatric Genomics Consortium (PCG‐ED). The Freeze 2 AN sample (*n* = 16,992 AN cases and 55,525 controls) (Watson et al., 2019) was used, with Australian and New Zealander participants (*n* = 2,536 AN cases and 15,624 controls) excluded to avoid potential crossover between base and target datasets. PLINK version 1.9 was used to calculate PRS at eight different *p* value thresholds (*p* < 5 × 10^−8^, *p* < 1 × 10^−5^, *p* < .001, *p* < .01, *p* < .05, *p* < .1, *p* < .5, *p* < 1.0) (Chang et al., 2015). The *p* value threshold that most strongly predicted the disordered eating outcome of interest in a previous study in this sample (Curtis et al., 2024) was used. This included *p* < .5 (global EDE, avoidance of eating), *p* < .05 (driven exercise) and *p* < .001 (objective bulimic episodes, self‐induced vomiting).

Interaction effects of PRS scores and environmental variables were tested using genome‐wide complex trait analysis (GCTA) genome‐based restricted maximum likelihood (GREML) analyses (Yang, Lee, Goddard, & Visscher, 2011). The interaction between the most predictive AN PRS scores and each environmental variable (parental expectations, parental criticism, parental conflict, parental care, weight‐related peer teasing) was tested against each disordered eating outcome (global EDE scores, avoidance of eating, objective bulimic episodes, driven exercise), totaling 25 analyses. Covariates included the first five principal components (PCs), and participant age and BMI centile at the same data collection time point as their highest recorded global EDE score. Covariates were controlled for by including each covariate, as well as all covariate × PRS and covariate × environment interaction terms in each model (Keller, 2014). Genetic relationship matrices (GRM), which estimate genetic relatedness between individuals based on single nucleotide polymorphisms (SNPs), were included to control for relatedness in the twin sample (Yang, Lee, Goddard, & Visscher, 2013). PRS scores were standardized (*M* = 0, *SD* = 1) and age, BMI centile, principal components, and all environmental measures were mean‐centered prior to analysis. To correct for multiple testing, the false discovery rate (FDR) adjusted *p* values (Q) were calculated using the Benjamini and Hochberg method (Benjamini & Hochberg, 1995). Results are reported as the proportion of variance in disordered eating explained by the environmental factors and their interactions with AN PRS (*r* ^2^).

## Results

Participants had a mean highest global EDE score of 0.73 (*SD* = 0.90, range 0–4.83), with 11% of participants (*n* = 43) scoring above the threshold for a ‘normal’ EDE‐Q score based on Australian community norms (i.e. <1.81) (Mond, Hay, Rodgers, Owen, & Beumont, 2004). The highest EDE scores were reported by 36% of participants (*n* = 138) at Wave 1, 30% (*n* = 117) at Wave 2, and 33% (*n* = 128) at Wave 3. The presence of lifetime disordered eating behaviours was 5%, 4.2%, 1.8% and 7.8%, for avoidance of eating, objective bulimic episodes, self‐induced vomiting, and excessive exercise, respectively, reflecting norms observed in comparable populations (Aardoom, Dingemans, Slof Op't Landt, & Van Furth, 2012; Luce, Crowther, & Pole, 2008; Machado et al., 2014; Mond, Hay, Rodgers, & Owen, 2006). Participants reported a mean score of 2.30 (*SD* = 0.49, range 1–3.8) for parental expectations, 1.77 (*SD* = 0.46, range 1–3.25) for parental criticism, 1.86 (*SD* = 0.38, range 1–3) for parental conflict, and 2.37 (*SD* = 0.38, range 1.33–3) for parental care. The mean score on the measure of weight‐related peer teasing was 1.43 (*SD* = 0.57, range 1–3.63). Tests for gene–environment correlations between AN PRS and each environmental factor showed no significant associations after FDR correction (Table S1).

### Parental expectations

**Figure 1:**
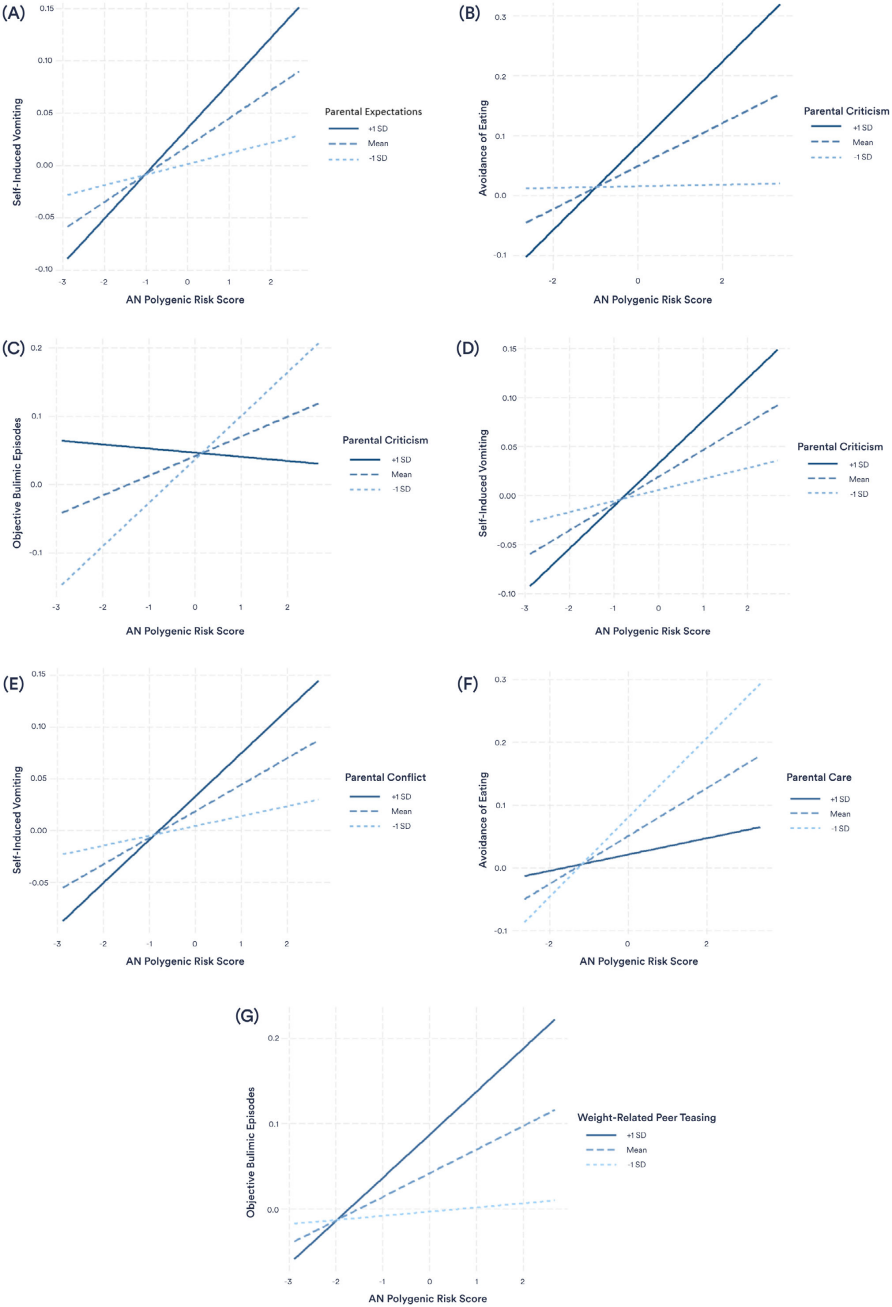
Interactions between environmental moderators and AN PRS in predicting disordered eating. (A) Parental expectations moderate the relationship between AN PRS and self‐induced vomiting. (B) Parental criticism moderates the relationship between AN PRS and avoidance of eating. (C) Parental criticism moderates the relationship between AN PRS and self‐induced vomiting. (D) Parental criticism moderates the relationship between AN PRS and objective bulimic episodes. (E) Parental conflict moderates the relationship between AN PRS and self‐induced vomiting. (F) Parental care moderates the relationship between AN PRS and self‐induced vomiting. (G) Weight‐related peer teasing moderates the relationship between AN PRS and objective bulimic episodes

There was a significant main effect of parental expectations on self‐induced vomiting (*r* ^2^ = 1.89%, *Q* = .025), with higher expectations associated with the presence of lifetime self‐induced vomiting (Table 2). Additionally, parental expectations moderated the relationship between AN PRS and self‐induced vomiting, with the association stronger in those who reported higher expectations (Figure 1A). The interaction between parental expectations and AN PRS scores explained 2.7% of variance in lifetime self‐induced vomiting (*Q* = .010). No other outcomes were significantly predicted by parental expectations or its interaction with AN PRS scores.

**Table 2:** Results of GREML analyses of parental factors and weight‐related peer teasing in predicting disordered eating outcomes, and their interactions with anorexia nervosa polygenic risk scores

Predictor	Outcome	Fixed effect	SE	*r* ^2^	*p*	*Q*
Parental expectations	Global EDE scores	0.18	0.09	0.0099	.053	.081
	Avoidance of eating	0.01	0.02	0.0007	.608	.633
	Objective bulimic episodes	0.04	0.02	0.0106	.052	0.081
	Self‐induced vomiting	0.04	0.01	0.0189	.008^a^	.025^b^
	Driven exercise	0.03	0.03	0.0026	.348	.421
Parental expectations × PRS	Global EDE scores	0.08	0.09	0.0020	.394	.462
	Avoidance of eating	−0.01	0.02	0.0010	.558	.591
	Objective bulimic episodes	−0.02	0.02	0.0023	.370	.441
	Self‐induced vomiting	0.04	0.01	0.0270	.002^a^	.010^b^
	Driven exercise	−0.02	0.03	0.0014	.514	.559
Parental criticism	Global EDE scores	0.48	0.10	0.0574	<.001^a^	<.001^b^
	Avoidance of eating	0.08	0.02	0.0258	.002^a^	.010^b^
	Objective bulimic episodes	0.00	0.02	0.0000	.853	.853
	Self‐induced vomiting	0.03	0.02	0.0074	.096	.134
	Driven exercise	0.04	0.03	0.0035	.267	.334
Parental criticism × PRS	Global EDE scores	0.19	0.10	0.0091	.056	.082
	Avoidance of eating	0.08	0.02	0.0272	.002^a^	.009^b^
	Objective bulimic episodes	−0.09	0.02	0.0435	<.001^a^	.001^b^
	Self‐induced vomiting	0.04	0.01	0.0194	.007^a^	.024^b^
	Driven exercise	−0.01	0.03	0.0000	.853	.853
Parental conflict	Global EDE scores	0.24	0.12	0.0099	.052	.081
	Avoidance of eating	0.06	0.03	0.0102	.058	.083
	Objective bulimic episodes	0.02	0.03	0.0010	.559	.591
	Self‐induced vomiting	0.04	0.02	0.0099	.053	.081
	Driven exercise	0.08	0.04	0.0128	.037^a^	.064
Parental conflict × PRS	Global EDE scores	0.23	0.12	0.0110	.054	.081
	Avoidance of eating	0.02	0.03	0.0019	.443	.503
	Objective bulimic episodes	0.05	0.03	0.0089	.099	.135
	Self‐induced vomiting	0.05	0.02	0.0185	.013^a^	.035^b^
	Driven exercise	0.06	0.04	0.0062	.151	.198
Parental care	Global EDE scores	−0.43	0.11	0.0334	<.001^a^	.002^b^
	Avoidance of eating	−0.07	0.03	0.0166	.013^a^	.034^b^
	Objective bulimic episodes	−0.02	0.03	0.0015	.458	.513
	Self‐induced vomiting	−0.02	0.02	0.0036	.244	.311
	Driven exercise	−0.03	0.04	0.0017	.432	.499
Parental care × PRS	Global EDE scores	−0.12	0.12	0.0027	.304	.374
	Avoidance of eating	−0.07	0.03	0.0147	.024^a^	.047^b^
	Objective bulimic episodes	0.02	0.03	0.0015	.471	.520
	Self‐induced vomiting	−0.03	0.02	0.0056	.157	.203
	Driven exercise	−0.06	0.04	0.0070	.133	.178
Weight‐related peer teasing	Global EDE scores	0.78	0.09	0.2449	<.001^a^	<.001^b^
	Avoidance of eating	0.01	0.02	0.0652	<.001^a^	<.001^b^
	Objective bulimic episodes	0.06	0.02	0.0271	.011^a^	.031^b^
	Self‐induced vomiting	0.03	0.02	0.0159	.052	.081
	Driven exercise	0.10	0.03	0.0497	<.001^a^	.004^b^
Weight‐related peer teasing × PRS	Global EDE scores	0.19	0.09	0.0134	.028^a^	.051
	Avoidance of eating	0.05	0.02	0.0150	.040^a^	.068
	Objective bulimic episodes	0.04	0.02	0.0219	.012^a^	.034^b^
	Self‐induced vomiting	0.02	0.01	0.0102	.088	.125
	Driven exercise	0.01	0.03	0.0004	.731	.751

*n =* 383. PRS, polygenic risk score for anorexia nervosa.

^a^ Significant with no FDR correction.

^b^ Significant after applying FDR correction.

### Parental criticism

The analyses revealed a significant main effect of parental criticism on both global EDE scores (*Q* = <.001) and avoidance of eating (*Q* = .010), with parental criticism explaining 5.74% and 2.58% of variance, respectively (Table 2). In both analyses, higher criticism was associated with higher disordered eating. A significant interaction was also found between parental criticism and AN PRS scores in predicting the presence of lifetime avoidance of eating (*r* ^2^ = 2.72%, *Q* = .009), with higher levels of parental criticism strengthening the association (Figure 1B). There was no main effect of parental criticism on objective bulimic episodes, self‐induced vomiting, or driven exercise, but analyses revealed a moderating effect of parental criticism on the relationship between AN PRS scores and both objective bulimic episodes and self‐induced vomiting. Higher parental criticism increased the influence of AN PRS scores on self‐induced vomiting (Figure 1C), whereas higher criticism decreased the effect of AN PRS on objective bulimic episodes (Figure 1D). The interactions explained 4.35% of variance in objective bulimic episodes (*Q* = <.001) and 1.94% of variance in self‐induced vomiting (*Q* = .024).

### Parental conflict

Parental conflict did not show a significant main effect on any disordered eating outcomes in the interaction model; however, there was a significant interaction between parental conflict and AN PRS scores in the prediction of self‐induced vomiting (Figure 1E). The association between AN PRS and self‐induced vomiting was stronger in participants who reported more parental conflict, with the interaction explaining 1.85% of variance (*Q* = .035) (Table 2).

### Parental care

Significant main effects were found for the relationship between maternal care and global EDE scores (*r* ^2^ = 3.34%, *Q* = .002), and avoidance of eating (*r* ^2^ = 1.66%, *Q* = .034) (Table 2). In both analyses, higher maternal care was associated with a decrease in the disordered eating outcomes. The interaction between maternal care and AN PRS also significantly predicted avoidance of eating, explaining 1.47% of phenotypic variance (*Q* = .047). AN PRS had a greater effect on avoidance of eating in participants who reported lower levels of maternal care (Figure 1F). The results for paternal care main effects reflected the maternal care effects; however, no significant interaction effects were found (Table S2).

### Weight‐related peer teasing

In our sample, weight‐related peer teasing was a significant predictor of global EDE scores (*r* ^2^ = 24.49%, *Q* = <.001), avoidance of eating (*r* ^2^ = 6.52%, *Q* = <.001), objective bulimic episodes (*r* ^2^ = 2.71%, *Q* = .031), and driven exercise (*r* ^2^ = 4.97%, *Q* = .004) (Table 2). In all analyses, higher weight‐related peer teasing predicted increased disordered eating outcomes. Higher weight‐related peer teasing was also found to moderate the relationship between AN PRS and lifetime objective bulimic episodes (Figure 1G). After applying the FDR correction, the interaction between weight‐related peer teasing and AN PRS significantly predicted objective bulimic episodes, explaining 2.19% of variance (*Q* = .034).

## Discussion

Building on our previous study (Curtis et al., 2024), which demonstrated that AN PRS predicted various disordered eating outcomes in an Australian adolescent female twin cohort, the present study explored the moderating role of environmental factors in this relationship. Specifically, we investigated whether weight‐related peer teasing or parental expectations, criticism, conflict, control, or care interacted with AN PRS to either increase the influence of AN PRS on disordered eating or serve as a protective factor for those at increased genetic risk.

Largely consistent with existing literature, EDE global scores were associated with higher levels of parental criticism and weight‐related peer teasing, and lower levels of parental care (Canetti, Kanyas, Lerer, Latzer, & Bachar, 2008; Eun, Paksarian, He, & Merikangas, 2018; Fairburn et al., 1997, 1998, 1999; Fairweather‐Schmidt & Wade, 2015, 2017; Micali et al., 2017; Neumark‐Sztainer et al., 2000; Wade et al., 2007; Wade & O'Shea, 2015). Of interest, these three variables were differentially associated with avoidance of eating; only weight‐related peer teasing was associated with objective bulimic episodes and driven exercise, and parental expectations were the only variable associated with self‐induced vomiting. Parental conflict, despite some support from previous research (Blodgett Salafia et al., 2014; Fairburn et al., 1999; Wade et al., 2001, 2007), showed no main effect on disordered eating. Weight‐related peer teasing was the most robust predictor of disordered eating, reflecting the impact of peer interactions and the declining influence of parents during adolescence (Klump et al., 2010). This suggests that targeting weight‐related peer teasing, including social media interactions, may be the most profitable target for eating disorder prevention and intervention.

Significant interactions were found between parental expectations, criticism, conflict, and care, and weight‐related peer teasing, and AN PRS in relation to specific disordered eating outcomes. While gene–environment correlations can potentially confound gene–environment interaction analyses, our analyses found no evidence of such correlations after FDR correction, supporting the validity of the interaction effects observed. As expected in a community sample, endorsement rates for the disordered eating outcomes were low, so all associations should be interpreted conservatively.

Higher parental expectations moderated the effect of AN PRS by further increasing the risk for self‐induced vomiting, and higher parental criticism interacted with AN PRS to increase the risk for both avoidance of eating and self‐induced vomiting. However, among participants with a higher AN PRS, higher parental criticism decreased the risk for objective bulimic episodes. One possible explanation for this is that the binge‐eating behaviour captured in the assessment of objective bulimic episodes differs from the restrictive tendencies assessed in avoidance of eating and self‐induced vomiting. Greater parental criticism may reinforce restrictive behaviours, which could, in turn, reduce the likelihood of binge eating episodes. However, given the low proportion of participants who reported lifetime objective bulimic episodes in the sample (4.2%, *n* = 16), this finding should be interpreted with caution. Replication in larger cohorts is required to clarify this relationship. To our knowledge, only one other study has explored the moderating effect of parental criticism on genetic risk for eating disorders. Karwautz et al. (2011) reported a significant moderating effect of higher parental criticism and parental control; however, the study only included participants with AN, and the interaction was explored with a candidate gene approach rather than a PRS, as in our study, so findings are not directly comparable (Karwautz et al., 2011).

Although parental conflict did not show a significant main effect on any disordered eating outcomes, it did moderate the relationship between AN PRS and self‐induced vomiting, with AN PRS having a stronger influence on disordered eating behaviours in individuals exposed to more parental conflict. Additionally, AN PRS had a stronger relationship with avoidance of eating in those who reported lower levels of parental care, suggesting that higher parental care may help to reduce the risk of disordered eating behaviours in individuals with genetic vulnerability. This suggests that polygenic risk may be “enhanced” by specific environmental risk and protective factors, demonstrating a clear point of intervention and potential environmental modification. These findings underscore the usefulness of family based therapy (FBT) for children and adolescents with AN where parent relationships are not treated as causal, but as factors that, when addressed, can improve treatment outcomes (Lock et al., 2015).

Finally, the results showed a significant interaction effect between weight‐related peer teasing and AN PRS in predicting objective bulimic episodes. This is notable as it supports previous work by Fairweather‐Schmidt and Wade (2017) using the same twin sample where they demonstrated a behavioural genetic gene–environment interaction with weight‐related peer teasing, whereby an increase in teasing strengthened the influence of genetic factors on disordered eating. To our knowledge, this is the first study to carry over a behavioural genetic finding to molecular genetic methods in this way. Although objective bulimic episodes were the only disordered eating outcome to be significantly predicted by this interaction, it is notable that prior to applying the FDR correction, weight‐related peer teasing also significantly moderated the effect of AN PRS on both global EDE scores and avoidance of eating. Given the community population of our sample, and the resulting low prevalence of disordered eating, this indicates a need for further investigation in larger cohorts to increase statistical power.

The present study had several strengths, including the application of polygenic risk scores and use of robust psychometric measures, including an interview‐based measure of disordered eating. In addition, investigating a general population sample was a strength, as it increases the generalisability of the findings to a broader population. However, the results should also be interpreted within the context of the following limitations. First, although this study used data obtained from a longitudinal study, data were analysed cross‐sectionally to maximise statistical power. Although disordered eating measures were taken from the wave of data collection where each participant was most affected to maximise power, and environmental measures were taken from the first wave of data collection to ensure these were reported either prior to or concurrent with the measure of disordered eating, this design means we cannot conclude whether the environmental factors are risk factors for disordered eating. However, detecting these environmental correlates is still useful and can help identify important factors to address in disordered eating interventions (Jacobi & Fittig, 2010).

It is important to note that the PRS scores used in the present study were likely underpowered due to the sample size of the AN GWAS used to generate the PRS (Watson et al., 2019). The amount of variance explained by the interactive models was also small, though this is typical of genetic models, as demonstrated by Watson et al.'s (2019) GWAS where the PRS explained 1.7% of variance in clinical AN cases. Additionally, the overall prevalence of disordered eating in our sample was low, although this reflected norms in comparable populations (Aardoom et al., 2012; Luce et al., 2008; Machado et al., 2014; Mond et al., 2006). Future studies with larger cohorts and increased statistical power may detect stronger or additional effects. Additionally, we were unable to control for pubertal development, which is associated with disordered eating aetiology (Klump, 2013), as the only available measure (menstruation status) showed insufficient variation in our sample to provide adequate statistical power (Wade et al., 2008). Another limitation is that the PRS were derived from AN GWAS data only, which may have influenced the pattern of our findings. Future research using PRS for other eating disorders may demonstrate different or additional genetic effects. Finally, participants in this study were adolescent females of European ancestry and available AN GWAS data is currently restricted to individuals of European ancestry, limiting generalisability of these findings to other demographics.

Overall, the present study demonstrates the complex interplay between genetic factors and environmental influences, particularly parental and peer interactions, in the development of disordered eating. Our findings have important clinical implications for disordered eating intervention, suggesting that prevention efforts should focus on promoting positive peer interactions, and that improving family dynamics by addressing parent relationships could lead to better treatment outcomes for genetically vulnerable individuals.

## Ethical information

Ethical approval was provided by the Flinders University Clinical Research Ethics Committee (no. 115/07) and written informed consent was obtained from all participants.

**Key points**

- Both genetic and environmental factors, and their interactions, contribute to disordered eating development, but research remains limited.
- We investigated how genetic risk for anorexia nervosa (AN), indicated by polygenic risk scores (PRS), interacts with environmental factors to contribute to disordered eating risk.
- Parental expectations, criticism, conflict, care and weight‐related peer teasing moderated the effect of AN PRS on disordered eating outcomes.
- Our results demonstrate the complex interplay between genetic and environmental factors in disordered eating development, specifically highlighting the need for personalized interventions that consider parental relationships and peer dynamics.

## Supporting information


**Table S1.** Results of GREML analyses testing gene‐environment correlations between the environmental factors and anorexia nervosa polygenic risk scores.
**Table S2.** Results of GREML analyses of paternal care in predicting disordered eating outcomes, and its interactions with anorexia nervosa polygenic risk scores.

## Data Availability

The data that support the findings of this study are available on request from the corresponding author. The data are not publicly available due to privacy or ethical restrictions.
