# Supplementary material for: Predicting adolescent disordered eating and behaviours: exploring environmental moderators of polygenic risk
Source: J Child Psychol Psychiatry. 2025 Jul 9;66(12):1795–806. doi: 10.1111/jcpp.70012 (PMC12626179; doi:10.1111/jcpp.70012)
Supplement: Supplementary file 1 — Table S1. Results of GREML analyses testing gene‐environment correlations between the environmental factors and anorexia nervosa polygenic risk scores. Table S2. Results of GREML analyses of paternal care in predicting disordered eating outcomes, and its interactions with anorexia nervosa polygenic risk scores. [file JCPP-66-1795-s001.docx]

**Supplementary Table 1**

Results of GREML Analyses Testing Gene-Environment Correlations between the Environmental Factors and Anorexia Nervosa Polygenic Risk Scores.

| *P*-value threshold | Environmental factor | *r^2^* | *p* | *Q* |
| --- | --- | --- | --- | --- |
| *p* < 0.001 | Parental expectations | 0.0001 | .848 | .954 |
|  | Parental criticism | 0.0005 | .658 | .954 |
|  | Parental conflict | 0.0018 | .419 | .954 |
|  | Parental care | 0.0003 | .738 | .954 |
|  | Weight-related peer teasing | 0.0004 | .692 | .954 |
| *p* < 0.05 | Parental expectations | 0.0111 | .040 | .302 |
|  | Parental criticism | 0.0000 | .898 | .954 |
|  | Parental conflict | 0.0001 | .892 | .954 |
|  | Parental care | 0.0001 | .845 | .954 |
|  | Weight-related peer teasing | 0.0003 | .739 | .954 |
| *p* < 0.5 | Parental expectations | 0.0174 | .011 | .168 |
|  | Parental criticism | 0.0059 | .143 | .717 |
|  | Parental conflict | 0.0000 | .954 | .954 |
|  | Parental care | 0.0013 | .496 | .954 |
|  | Weight-related peer teasing | 0.0013 | .450 | .954 |

**Supplementary Table 2**

Results of GREML Analyses of Paternal Care in Predicting Disordered Eating Outcomes, and its Interactions with Anorexia Nervosa Polygenic Risk Scores.

| Predictor | Outcome | Fixed effect | SE | *r^2^* | *p* | *Q* |
| --- | --- | --- | --- | --- | --- | --- |
| **Paternal care** | Global EDE scores | -0.38 | 0.11 | 0.0361 | <.001* | .007** |
|  | Avoidance of eating | -0.08 | 0.03 | 0.0275 | .003* | .020** |
|  | Objective bulimic episodes | -0.00 | 0.02 | 0.0000 | .959 | .959 |
|  | Self-induced vomiting | -0.01 | 0.01 | 0.0024 | .361 | .387 |
|  | Driven exercise | -0.03 | 0.03 | 0.0031 | .319 | .368 |
| **Paternal care x PRS** | Global EDE scores | -0.13 | 0.10 | 0.0047 | .227 | .295 |
|  | Avoidance of eating | -0.05 | 0.03 | 0.0125 | .053 | .113 |
|  | Objective bulimic episodes | 0.03 | 0.03 | 0.0045 | .236 | .295 |
|  | Self-induced vomiting | -0.02 | 0.01 | 0.0050 | .204 | .295 |
|  | Driven exercise | -0.06 | 0.03 | 0.0106 | .081 | .150 |

*Note. n =* 372. PRS = polygenic risk score for anorexia nervosa. *Significant with no FDR correction, **significant after applying FDR correction.
